# Idiopathic Tumoral Calcinosis in Hand: A Case Report

**Published:** 2014-07-24

**Authors:** Chris Xu, Josephine Alexa Potter, Christopher David Carter, Clayton Miles Cooper Lang

**Affiliations:** ^a^Department of Plastic and Reconstructive Surgery, The Queen Elizabeth Hospital, Woodville South, South Australia, Australia; ^b^Department of Anatomical Pathology, SA Pathology and Department of Pathology, Flinders University, South Australia, Australia

**Keywords:** hand, tumoral calcinosis, surgery, idiopathic, calciphylaxis

## Abstract

**Objective:** Tumoral calcinosis is an uncommon lesion, composed of ectopic calcified tissue, most commonly seen in the large joints of the hips, knees, shoulders, and elbows. The involvement of the hand in a healthy patient is extremely rare, and therefore this condition can cause diagnostic confusion. The purpose of this report is to describe one case of idiopathic tumoral calcinosis that occurred in the left hand of a 35-year-old healthy female patient. **Methods:** The patient presented with 2-day history of acutely swelling and painful left hand middle finger metacarpal phalangeal joint without any precipitants. **Results:** All biochemical, radiological, and histopathological evidence suggested idiopathic tumoral calcinosis of the hand. **Conclusions:** In this case, surgery provided the patient with instant symptomatic relief and full functional recovery of that joint.

Tumoral calcinosis is an uncommon benign condition characterized by large calcified periarticular soft tissue masses composed of calcium salts, usually located around large joints. The exact etiology remains unknown. The disease was first recognized in 1898 by Giard, and then in 1899 by Duret, who reported 2 cases occurring in 2 siblings and named the process endothelium calcifie. In 1935, Teutschlaender, who investigated the disease for more than 20 years, labeled the lesion lipocalcinogranulomatosis (Teutschlaender disease). Unaware of this previous work, Inclan in 1943 described 3 patients with large periarticular calcified masses, which he called tumoral calcinosis. This name has been accepted by international community since then.^1^^,^^2^^,^^5^^-^7

There is a primary form (idiopathic or hereditary), but tumoral calcinosis is often found in a wide variety of conditions secondary to hyperparathyroidism, vitamin D intoxication, scleroderma, and uremia in the context of chronic renal failure.

## CASE PRESENTATION

**Figure 1:**
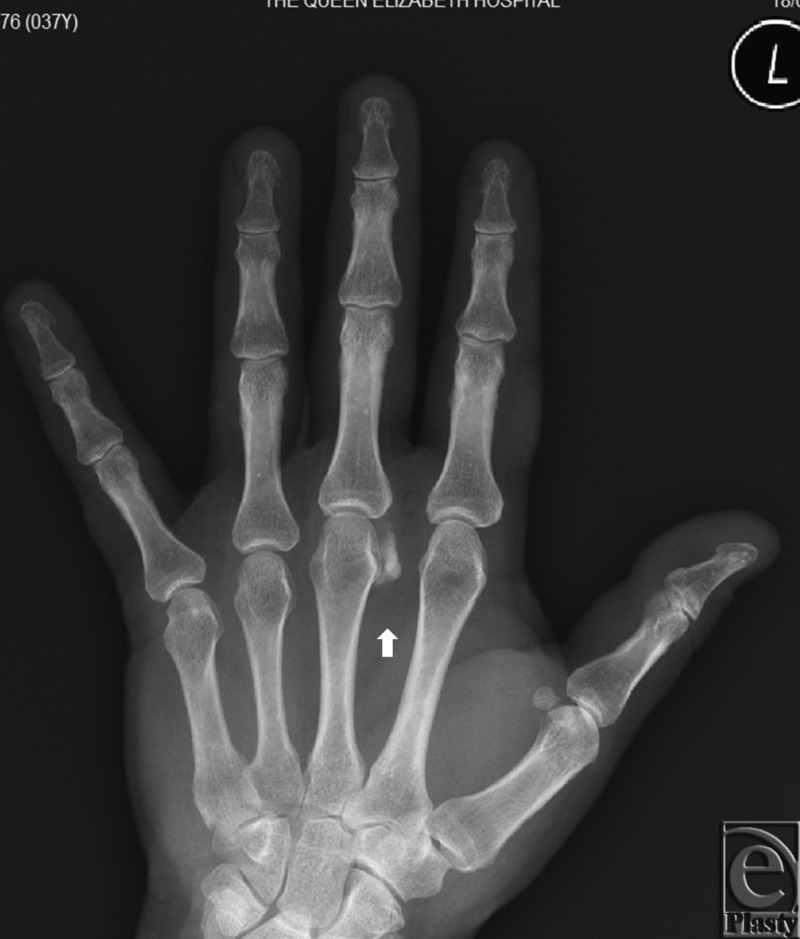
X-ray. Single 12 mm × 4 mm radio-opaque mass (arrow) can be visualized alongside the radial border of the affected joint.

**Figure 2:**
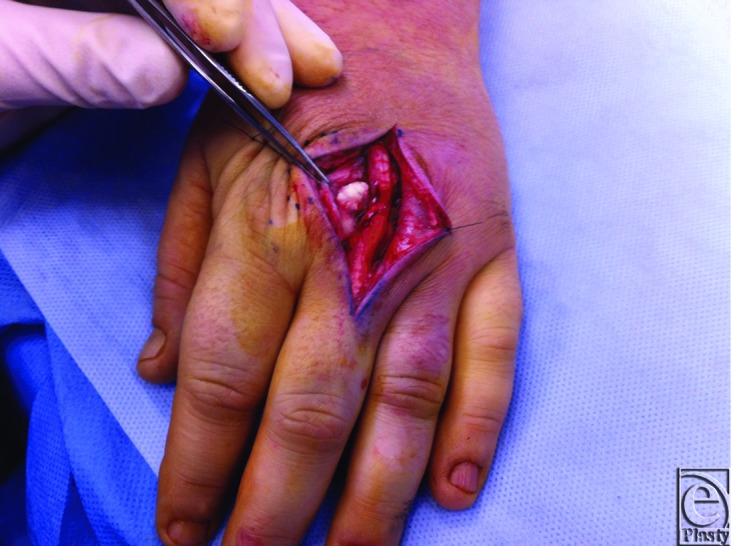
Intraoperative aspect. Single white tophus-like structure was excised. The structure was adjacent to metacarpophalangeal joint, but not penetrating into the joint capsule. Some collateral ligament attachment was observed.

**Figure 3:**
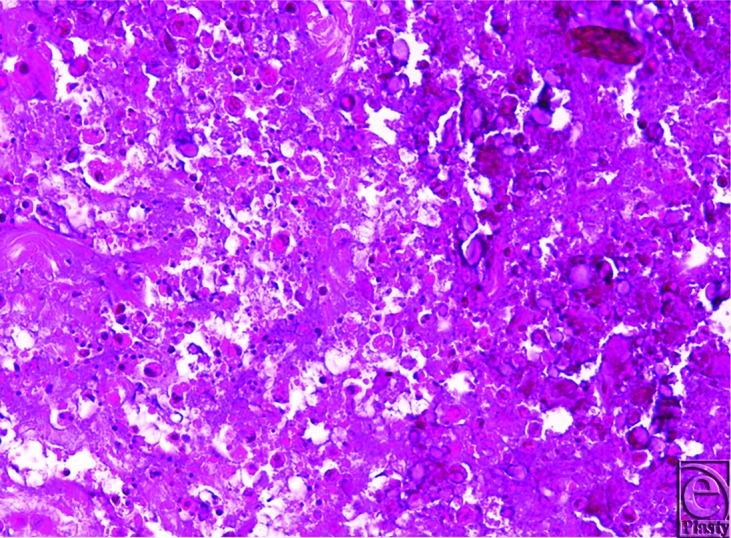
Histopathological aspect. There is amorphous calcific material with an infiltrate of neutrophils and macrophages.

A 35-year-old right hand dominant housewife presented to The Queen Elizabeth Hospital emergency department with spontaneous onset with 2-day history of swollen and tender left middle finger metacarpophalangeal joint. No history of trauma was recalled. She is a smoker and had laparoscopic cholecystectomy many years ago secondary to cholelithiasis. On further questioning, she reported a similar episode 2 weeks prior at the same joint. However, the symptoms settled within 24 hours and did not prompt her to seek medical attention. On examination, the affected joint was swollen, exquisitely painful on passive movement, erythematous, and warm to touch. Biochemical study showed normal calcium, phosphate, parathyroid hormone, vitamin D, and thyroid function level. X-ray showed a prominent area of soft tissue calcification projected just dorsolateral to the head of the middle finger metacarpal bone measuring 12 mm × 4 mm in diameter (Fig 1). Ultrasound-guided biopsy was unable to aspirate any sample due to the relative solid nature of the lesion. Initial differential diagnosis was either a crystalline arthropathy or early septic arthritis and empirical therapy (intravenous cefazolin and oral colchicine) for both conditions were commenced. Minimal improvement was observed overnight, and the decision was therefore made to perform an arthrotomy and irrigation of the joint. Perioperatively, access to the lesion was achieved by a single curvilinear dorsal incision. A single gouty tophus like structure was found external to the joint, but infiltrating into the collateral ligaments of the joint (Fig 2). Sharp dissection was done from paratenon to joint capsule. The lesion was excised and deposits removed from collateral ligament as much as possible. There was no evidence of ulceration or fistula formation with the superficial skin. Histology showed a circumscribed ovoid mass of amorphous granular calcific debris containing sparse macrophages and lymphocytes and having a peripheral rim of dense fibrotic tissue, typical of tumoral calcinosis at an inactive late stage. In addition, the calcific deposit contained a moderate neutrophilic inflammatory infiltrate (Fig 3). No crystals or bacteria were identified in the joint aspirate specimen.

## DISCUSSION

Tumoral calcinosis is an uncommon form of ectopic calcification characterized by large, rubbery, or cystic masses occurring mainly in relation to large joints. Prior studies have suggested that tumoral calcinosis most commonly occurs around the hip, shoulder, and elbow while it is rare in the small joints of the hand and foot.^1^^-^4

Primary tumoral calcinosis should be differentiated from secondary tumoral calcinosis, which occurs in association with renal failure and with hypercalcemic disorders. It has been suggested by Smack et al, tumoral calcinosis should be grouped into 3 categories based on pathogenesis. The primary familial form of the disease mainly affects young, African males with elevated serum phosphate and vitamin D. The identified major genes responsible for the autosomal recessive pattern of inheritance are *GALNT3*, *FGF-23*, and *Klotho*, 3 genes whose proteins are important in promoting phosphate excretion and suppressing vitamin D synthesis.^2^ Patients frequently present with multiple tumoral calcinosis affecting multiple joints, with a high recurrence rate after surgical excision. In contrast, the primary idiopathic form of the disease affects patients who have no family history of the condition or known disorders of phosphate or calcium metabolism. In this instance, tumoral calcinosis is normally a single event with low incidence of recurrence.^4^^,^^7^

Finally, a secondary form of tumoral calcinosis results from a concurrent disease that is capable of causing excessive calcium deposition conditions such as chronic renal failure ± dialysis, hyperparathyroidism, vitamin D intoxication, milk alkali syndrome, collagen vascular disease (ie, scleroderma), and bony destruction secondary to malignancy.^2^ Secondary tumoral calcinosis is best controlled by effective management of the primary underlying condition.

Tumoral calcinosis has a typical appearance of amorphous, cystic, and multilobulated periarticular calcification on plain radiographs. Tumoral calcinosis in the large joints is often reported as a dense “chicken wire” type of lucencies that give the calcification a “cobblestone” appearance. Computed tomography delineates the calcific mass, whereas the fluid level seen within is termed a “sedimentation sign.” Similar findings can be found in magnetic resonance imaging as a hypointense-dependent layer on T2 images. Erosions and osseous destruction by the adjacent soft tissue mass is usually absent.^7^^,^^8^ These findings help to distinguish tumoral calcinosis from other diseases causing periarticular soft tissue calcification such as synovial osteochondromatosis, neoplasms, connective tissue diseases, or degenerative changes.^7^^,^^8^ Our case's hand X-ray showed the lesion to be a rounded, dense, periarticular opacity that was typical of tumoral calcinosis of the hand. It was relatively homogenous, which is suggestive of a reduced metabolic activity with a reduced chance of growth. Tumoral calcinosis is frequently recognized in preoperative imaging before definitive histological examination.^3^

Histologically, periarticular calcification is an obligatory finding for tumoral calcinosis.^7^ Other diagnostic criteria for this condition are the amorphous granular nature of the calcific material and the absence of crystal deposits. An unusual finding in our case was the presence of a moderate neutrophilic inflammatory infiltrate within the calcific debris, raising the possibility of superimposed infection. However, other case reports describe a mild neutrophilic component found on histology, without evidence of an infective component.^1^ This acute inflammatory response in our case may simply relate to traumatization of the lesion although there remains the possibility of sepsis, particularly in view of the preoperative needle aspiration procedure. Cartilage and synovium are not normally found in the lesion itself in cases of tumoral calcinosis.

The treatment of tumoral calcinosis should be tailored to the clinical setting. For patients with secondary tumoral calcinosis, managing the underlying calcium/phosphate homeostasis is the primary goal. In patients with end-stage renal failure, it is important to differentiate secondary tumoral calcinosis from calciphylaxis due to high mortality rate of the latter one. The clinical presentation and radiological and histological appearances of calciphylaxis significantly differ from secondary tumoral calcinosis. Clinically, patients with calciphylaxis develop superficial violaceous skin lesions that are painful, hyperesthetic, or pruritic. More widespread ischemia can produce a mottled or serpiginous skin appearance similar to livedo reticularis. With tissue infarction, the lesions become intensely necrotic, hemorrhagic, and painful and spread to contiguous areas. Radiologically, calciphylaxis presents with small and medium vessel calcifications, and a netlike pattern of soft tissue calcifications. Histologically, calciphylaxis is characterized by microvascular medial calcification and intimal hypertrophy associated with cutaneous ischemia and ulceration.^9^ Overall, calciphylaxis can be confidently diagnosed clinically and from skin biopsy.

Surgical excision of tumoral calcinosis is indicated if there is significant skin breakdown or joint involvement. Early excision should be undertaken while the masses are small to avoid operative difficulties and ulceration of the overlying skin with secondary infection that occur with very large masses.^6^ Excision may be difficult if no true capsule is present as the mass may invade adjacent muscle tissue. Recurrence in idiopathic tumoral calcinosis is uncommon. Spontaneous regression of the mass of tumoral calcinosis has never been reported. The prognosis is good particularly in the absence of secondary infection.

Increased awareness of tumoral calcinosis is essential. Complete surgical resection is the treatment of choice in most idiopathic cases. Alternative treatments using radiotherapy and systemic steroids have been unsuccessful.^7^ Finally, the role of the surgeon consists not only in the proper excision of the mass but also in reconstruction of any resulting tissue defect or functional deficit. With the additional help of early hand therapy, complete functional return of the affected digits should be expected.
